# Predicting Adverse Outcomes in Upper Gastrointestinal Bleeding: A Focus on Blood Urea Nitrogen‐Based Ratios and Age‐Adjusted Shock Index

**DOI:** 10.1155/emmi/3772312

**Published:** 2026-07-08

**Authors:** Omer Faruk Cakiroglu, Julide Gurbuz, Bilal Arac, Yilmaz Ersoz, Bahadir Taslidere, Basar Cander

**Affiliations:** ^1^ Department of Emergency Medicine, Kartal Doktor Lutfi Kirdar City Hospital, Istanbul, Türkiye; ^2^ Department of Emergency Medicine, Bezmialem Vakif University, Istanbul, Türkiye, bezmialem.edu.tr; ^3^ Department of Emergency Medicine, Basaksehir State Hospital, Istanbul, Türkiye; ^4^ Department of Emergency Medicine, Sancaktepe Sehit Ilhan Varank Training and Research Hospital, Istanbul, Türkiye

**Keywords:** age-adjusted shock index, BUN/haemoglobin ratio, BUN/platelet ratio, intensive care, mortality, risk stratification, upper gastrointestinal bleeding

## Abstract

**Background/Aim:**

Rapid risk stratification is crucial in managing upper gastrointestinal bleeding (UGIB). This study evaluated the prognostic value of the age‐adjusted shock index (ASI), blood urea nitrogen (BUN)/haemoglobin ratio and BUN/platelet ratio for predicting adverse clinical outcomes.

**Materials and Methods:**

This single‐centre retrospective observational cohort study included 204 adult patients (age ≥ 18 years) presenting to the emergency department with UGIB between 1 January 2022 and 31 December 2022. Patients with oesophageal variceal bleeding, lower gastrointestinal sources, trauma, pregnancy, malignancy, prior diagnosis at another institution or incomplete data were excluded. Receiver operating characteristic (ROC) curve analysis was the primary statistical method used to determine prognostic performance.

**Results:**

Among the 204 included patients, the intensive care unit (ICU) admission rate was 14%, and the in‐hospital mortality rate was 5.4%. ROC curve analyses for in‐hospital mortality revealed statistically significant predictive capabilities across all evaluated parameters. The BUN/haemoglobin ratio demonstrated an area under the curve (AUC) of 0.779 at an optimal cut‐off of > 8.45, yielding a high negative predictive value (NPV) of 97.26%. The BUN/platelet ratio produced an AUC of 0.747 at a cut‐off of > 0.17, with an NPV of 98.32%. Additionally, the ASI achieved an AUC of 0.780 at an optimal cut‐off of > 61.72 and an NPV of 98.03%.

**Conclusion:**

The ASI, BUN/haemoglobin and BUN/platelet ratios are rapid, inexpensive and easily calculable parameters upon admission. Their notably high NPVs make them effective complementary tools to established scoring systems, aiding clinicians in safely identifying low‐risk patients who can be managed in the general ward rather than the ICU.

## 1. Introduction

As an indicator, the blood urea nitrogen (BUN)/haemoglobin ratio concurrently reflects two physiological mechanisms: volume loss and decreased renal perfusion [3, 4]. In massive haemorrhages, a substantial volume of blood rapidly enters the bowel lumen, leading to a prompt elevation in BUN levels. In minor bleeds, even if prolonged, the presence of blood in the bowel lumen is reduced, consequently decreasing the amount absorbed. This pathophysiological approach underpins the diagnostic and prognostic value of the BUN/haemoglobin ratio in massive bleeding scenarios [5].

The platelet count is not included in the majority of widely used upper gastrointestinal bleeding (UGIB) risk scores, and its prognostic value in patient populations other than those with cirrhotic or variceal bleeding remains to be clearly established [6]. Cohort studies have demonstrated that thrombocytopenia increases the risk of gastrointestinal bleeding and subsequent mortality; however, the isolated and combined prognostic value of the platelet count in acute UGIB has not been adequately investigated [7]. In this context, evaluating the platelet count in conjunction with biochemical markers such as BUN—which correlates with bleeding severity and intraluminal blood digestion—may yield additional and complementary prognostic information for risk stratification in UGIB.

The literature indicates that the shock index impacts mortality in patients with UGIB and should be considered during patient management decisions [8]. However, the specificity and sensitivity of the shock index may be diminished in elderly patients [9]. For this reason, the age‐adjusted shock index (ASI) has gained prominence. Calculated by multiplying the shock index by the patient’s age, the ASI accounts for age‐related cardiovascular changes and reduced haemodynamic reserve, particularly within geriatric populations. This provides a more sensitive assessment for adverse clinical outcomes in the event of bleeding [10, 11]. The Glasgow–Blatchford score (GBS) is a valuable tool for identifying low‐risk patients in UGIB; however, its ability to predict mortality is limited, as it primarily reflects the need for transfusion or intervention. In addition, the inclusion of multiple clinical and laboratory variables may limit its rapid and practical use in emergency settings [12]. AIMS65, on the other hand, mainly focuses on predicting mortality but demonstrates lower performance in identifying other clinically relevant outcomes such as the need for intervention [13]. One of the main limitations of the Rockall score is that its full version requires endoscopic findings, which restricts its applicability in the early phase of emergency department assessment; although the pre‐endoscopic version can be used, its performance remains limited for several outcomes [14]. In light of these limitations, novel parameters such as BUN‐based ratios and the ASI may help overcome some of the limitations of existing scoring systems. These indices can more practically reflect both the severity of bleeding and the patient’s physiological status. Their ease of calculation using fewer variables makes them particularly suitable for use in emergency settings. Furthermore, they may provide additional value in early risk stratification and in predicting poor outcomes. Therefore, these parameters may serve as complementary, and in some cases alternative, tools to conventional scoring systems.

In this study, we evaluated the association of the ASI, BUN/haemoglobin and BUN/platelet ratios, as well as AIMS‐65 and the GBS with mortality and intensive care unit (ICU) admission in patients with UGIB, and investigated the clinical utility of these parameters in daily practice.

## 2. Materials and Methods

**FIGURE 1:**
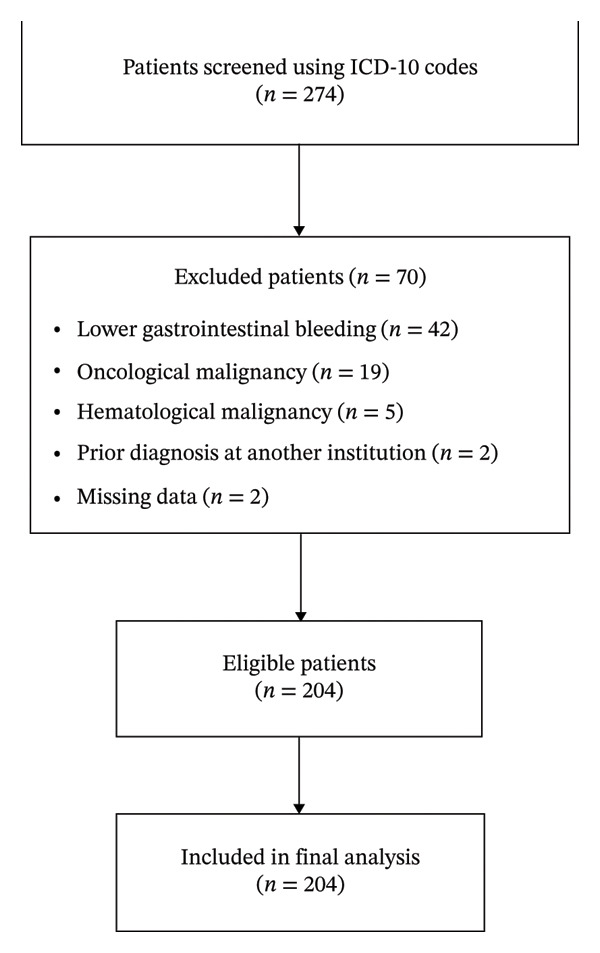
Flow diagram of patient selection.

This study was conducted as a single‐centre retrospective observational cohort study evaluating prognostic indicators in patients with UGIB, following approval from the institutional ethics committee. Adults aged ≥ 18 years who presented to the emergency department and were diagnosed with UGIB were eligible for inclusion. The UGIB diagnosis was established based on a combination of clinical presentation (haematemesis and melena), ICD‐10 coding, and endoscopic findings where available. A total of 274 patients were screened using ICD‐10 codes. After applying predefined exclusion criteria, 70 patients were excluded due to lower gastrointestinal bleeding (*n* = 42), oncological malignancy (*n* = 19), haematological malignancy (*n* = 5), prior diagnosis at another institution (*n* = 2), and missing data (*n* = 2). The final study cohort consisted of 204 patients (Figure 1). Identified patients were screened using ICD‐coded electronic records. Patients were evaluated according to predefined inclusion and exclusion criteria, and those meeting eligibility criteria were included in the study. Patients were identified via the hospital electronic information system using the ICD‐10 code K92 and were screened over a one‐year period from 1 January 2022 to 31 December 2022.

Patients who had received a diagnosis at another institution prior to transfer, those without UGI‐source bleeding, those with oesophageal variceal bleeding, trauma patients, pregnant patients, patients with malignancy and those with incomplete data were excluded. A comprehensive data collection form was developed, including patients’ registration numbers, age, sex, chronic comorbidities (hypertension and diabetes mellitus) and their severity, presenting complaints, symptoms (haematochezia, haematemesis, melaena, syncope and ongoing bleeding), digital rectal examination findings, blood pressure, baseline renal function tests (BUN and creatinine) and bleeding‐related laboratory parameters (prothrombin time [PT], activated partial thromboplastin time [aPTT], international normalised ratio [INR], haemoglobin, haematocrit and platelet count). Endoscopic findings, rebleeding during follow‐up, in‐hospital complications, disposition/outcomes (discharge, death, ward admission and ICU admission) and lengths of stay in the emergency department and hospital were also recorded.

The prognostic performance and association with mortality of the ASI, the BUN/haemoglobin ratio and the BUN/platelet ratio were analysed through comparative assessments. No formal a priori sample size calculation was performed because this was a retrospective cohort study based on ICD‐10‐screened eligible patients during a predefined study period. For the ICU admission multivariable model, the events‐per‐variable ratio was 14.5, which was consistent with commonly suggested recommendations for multivariable regression modelling. Statistical analyses were performed using SPSS Version 25.0 (IBM Corp., Armonk, NY, USA). The normality of continuous variables was assessed using histogram plots and the Kolmogorov–Smirnov test. Given that most continuous variables were not normally distributed, nonparametric tests were applied. Descriptive statistics were reported using mean, standard deviation (SD), median and minimum–maximum (min–max) values. Categorical variables were presented as *n* (%), and continuous variables as mean ± SD and/or median (min–max), as appropriate. Comparisons in 2 × 2 tables were performed using Pearson’s chi‐squared test. The Mann–Whitney *U* test was used for between‐group comparisons of nonnormally distributed variables. Receiver operating characteristic (ROC) curve analyses were conducted to calculate sensitivity, specificity, area under the curve (AUC) and optimal cut‐off values. Comparisons of AUCs between ROC curves were performed using the DeLong method. Associations between variables were assessed using Pearson or Spearman correlation analyses, with Pearson’s correlation used for normally distributed variables and Spearman’s correlation used for nonnormally distributed variables. Relationships between continuous variables and binary outcomes (mortality and ICU admission) were evaluated using point‐biserial correlation analysis. ASI, the BUN/haemoglobin ratio and the BUN/platelet ratio were compared between binary outcome groups and are reported with sensitivity, specificity, positive predictive value (PPV) and negative predictive value (NPV). All *p* values < 0.05 were considered statistically significant. Multivariable logistic regression analyses were conducted to assess the independent predictive value of the study parameters for ICU admission. Variables were selected based on univariable significance (*p* < 0.05) and clinical relevance. Clinically relevant confounders such as age, sex, comorbidities and vital signs were considered; however, due to the limited number of events, the final model was restricted according to the events‐per‐variable principle to avoid overfitting. Model assumptions were evaluated using the Box–Tidwell procedure, and multicollinearity was assessed using variance inflation factors (VIF). Internal validation was performed using 5000 bootstrap samples with bias‐corrected and accelerated (BCa) confidence intervals. Results were reported as odds ratios (OR) with 95% confidence intervals. Model calibration was assessed using the Hosmer–Lemeshow goodness‐of‐fit test (10 deciles) and visualised with calibration curves. Internal validation was further performed using 10‐fold cross‐validation to generate optimism‐corrected AUC estimates. Clinical utility was evaluated using decision curve analysis (DCA).

This study was reported in accordance with the STROBE reporting guidelines for observational studies and the TRIPOD recommendations for prognostic model reporting (Supporting Information 5).

## 3. Results

A total of 204 patients were included in the study, comprising 135 men and 69 women. The median age was 70 years (58–80). Regarding admission status, 29 patients (14%) were admitted to the ICU, and 175 (86%) to a general ward. The mean length of stay was 4 days. During the hospital course, 193 patients (94.6%) survived, while 11 (5.4%) were recorded as deceased. The median AIMS‐65 score was 1 (0.0–2.0), and the median Glasgow–Blatchford bleeding score was 10 (0.0–20.0) (Table 1).

**TABLE 1:** Demographic and clinical data.

Characteristic	*N* = 204^1^
Sex (male)	135 (66%)
Age (years)	70 (58, 80)
Temperature (°C)	36.10 (36.00, 36.50)
SpO_2_ (%)	97.00 (96.00, 98.00)
Heart Rate (beats/min)	92 (80, 109)
Systolic Blood Pressure (mmHg)	126 (104, 147)
Diastolic Blood Pressure (mmHg)	67 (±17.1)
Mean Corpuscular Volume (MCV) (fL)	88 (84, 93)
Haemoglobin (g/dL)	9.67 (7.41, 12.10)
Haematocrit (%)	31.1 (±8.62)
Platelet Count (× 10^3^/μL)	226 (181, 280)
Red Blood Cell Count (× 10^6^/μL)	3.45 (±1.02)
Blood Urea Nitrogen (BUN) (mg/dL)	32 (21, 47)
Creatinine (mg/dL)	1.02 (0.82, 1.27)
Sodium (Na^+^) (mmol/L)	138.0 (134.5, 140.0)
Potassium (K^+^) (mmol/L)	4.34 (4.02, 4.73)
Bicarbonate (HCO_3_^−^) (mmol/L)	24.0 (21.9, 25.3)
pH	7.40 (±0.05)
BUN/Haemoglobin Ratio	3.51 (1.88, 5.67)
BUN/Platelet Ratio (× 10^3^)	0.14 (0.09, 0.23)
Age‐Adjusted Shock Index	50 (40, 62)
AIMS‐65	1 (0.00–2.00)
Glasgow–Blatchford Bleeding Score	10 (0.00–2.00)
Length of Hospital Stay (days)	4.0 (3.0, 6.0)
Intensive Care Unit (ICU) Admission	29 (14%)
Mortality	11 (5.4%)

^1^Mean (±SD); median (Q1, Q3); *n* (%).

The median BUN/haemoglobin ratio was 4.27 (Q1–Q3: 2.86–8.54) in ICU patients and 3.41 (Q1–Q3: 1.76–5.29) in ward patients (*p* = 0.007). The median BUN/platelet ratio was 0.18 (Q1–Q3: 0.10–0.34) in ICU patients and 0.13 (Q1–Q3: 0.09–0.22) in ward patients (*p* = 0.130). The median ASI was 55 (Q1–Q3: 46–62) in ICU patients and 49 (Q1–Q3: 38–63) in ward patients (*p* = 0.039) (Table 2).

**TABLE 2:** Comparison of prognostic parameters according to ICU and ward admission.

	Ward *N* = 175^1^	ICU *N* = 29^1^	*p* value^2^
Length of Hospital Stay (days)	4.0 (3.0, 5.0)	9.0 (5.0, 13.0)	< 0.001
BUN/Haemoglobin Ratio	3.41 (1.76, 5.29)	4.27 (2.86, 8.54)	0.007
BUN/Platelet Ratio (× 10^3^)	0.13 (0.09, 0.22)	0.18 (0.10, 0.34)	0.130
Age‐Adjusted Shock Index	49 (38, 63)	55 (46, 62)	0.039
AIMS‐65	1 (0.0, 2.0)	1 (1.0, 3.0)	0.002
Glasgow–Blatchford Bleeding Score	10 (6.0, 13.0)	13 (10.0, 14.0)	0.004

^1^Median (Q1, Q3); *n*/*N* (%).

^2^Wilcoxon rank sum test; Students *t*‐test; Pearson’s chi‐squared test; Fisher’s exact test.

In the comparison between ward and ICU admission groups, the AIMS‐65 score was significantly higher in patients admitted to the ICU (1 [1.0–3.0] vs. 1 [0.0–2.0], *p* = 0.002). Similarly, the Glasgow–Blatchford score was also significantly higher in the ICU group (13 [10.0–14.0] vs. 10 [6.0–13.0], *p* = 0.004) (Table 2).

In the mortality analysis, the median BUN/haemoglobin ratio was 8.47 (Q1–Q3: 4.09–8.88) among patients who died and 3.43 (Q1–Q3: 1.81–5.36) among survivors; this difference was statistically significant (*p* = 0.002). For the BUN/platelet ratio, the median was 0.24 (Q1–Q3: 0.17–0.64) in nonsurvivors compared with 0.13 (Q1–Q3: 0.09–0.21) in survivors (*p* = 0.006). The median ASI was 69 (Q1–Q3: 56–78) in nonsurvivors and 49 (Q1–Q3: 39–61) in survivors (*p* = 0.002) (Table 3).

**TABLE 3:** Association between prognostic parameters and mortality.

Characteristic	Survivors *N* = 193^1^	Nonsurvivors *N* = 11^1^	*p* value^2^
Length of Hospital Stay (days)	4.0 (3.0, 6.0)	4.0 (1.0, 10.0)	0.800
BUN/Haemoglobin Ratio	3.43 (1.81, 5.36)	8.47 (4.09, 8.88)	0.002
BUN/Platelet Ratio (× 10^3^)	0.13 (0.09, 0.21)	0.24 (0.17, 0.64)	0.006
Age‐Adjusted Shock Index	49 (39, 61)	69 (56, 78)	0.002
AIMS‐65	1 (0.0, 2.0)	2 (1.5–3.0)	< 0.001
Glasgow–Blatchford Bleeding Score	10 (6.0–13.0)	12 (9.00–13.5)	0.125

^1^Median (Q1, Q3); *n*/*N* (%).

^2^Wilcoxon rank sum test; Students *t*‐test; Pearson’s chi‐squared test; Fisher’s exact test.

In the comparison between survivors and nonsurvivors, the AIMS‐65 score was significantly higher in nonsurvivors (2 [1.5–3.0] vs. 1 [0.0–2.0], *p* < 0.001). In contrast, the Glasgow–Blatchford score did not show a statistically significant difference between the two groups (12 [9.0–13.5] vs. 10 [6.0–13.0], *p* = 0.125) (Table 3).

**FIGURE 2:**
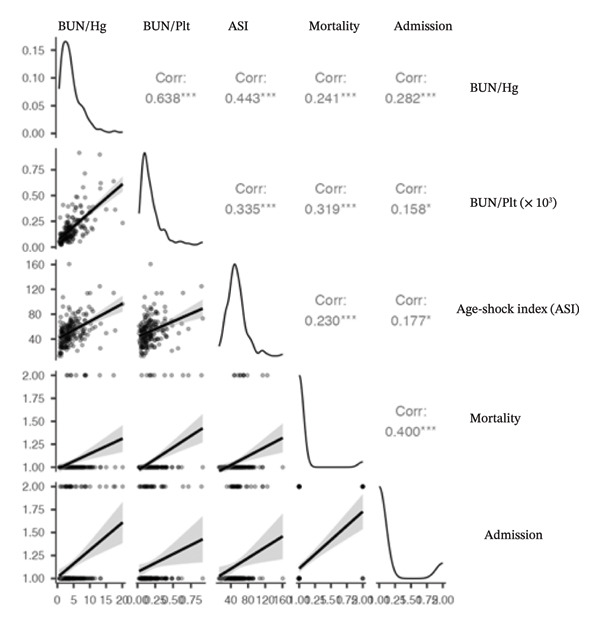
Correlation plot of prognostic parameters.

Pearson correlation analyses were performed to assess the relationships between the BUN/haemoglobin ratio, the BUN/platelet ratio and the ASI with in‐hospital mortality and ICU admission. A strong positive correlation was observed between the BUN/haemoglobin and BUN/platelet ratios (*r* = 0.638, *p* < 0.001). A moderate positive correlation was found between the BUN/haemoglobin ratio and ASI (*r* = 0.443, *p* < 0.001). Both the BUN/haemoglobin and BUN/platelet ratios showed weak correlations with in‐hospital mortality (*r* = 0.241, *p* < 0.001 and *r* = 0.319, *p* < 0.001, respectively) and with ICU admission (*r* = 0.283, *p* < 0.001 and *r* = 0.158, *p* = 0.024, respectively). For ASI, weak associations were identified with mortality (*r* = 0.230, *p* < 0.001) and ICU admission (*r* = 0.177, *p* = 0.011). Finally, a moderate and statistically significant correlation was observed between mortality and ICU admission (*r* = 0.400, *p* < 0.001) (Figure 2).

**FIGURE 3:**
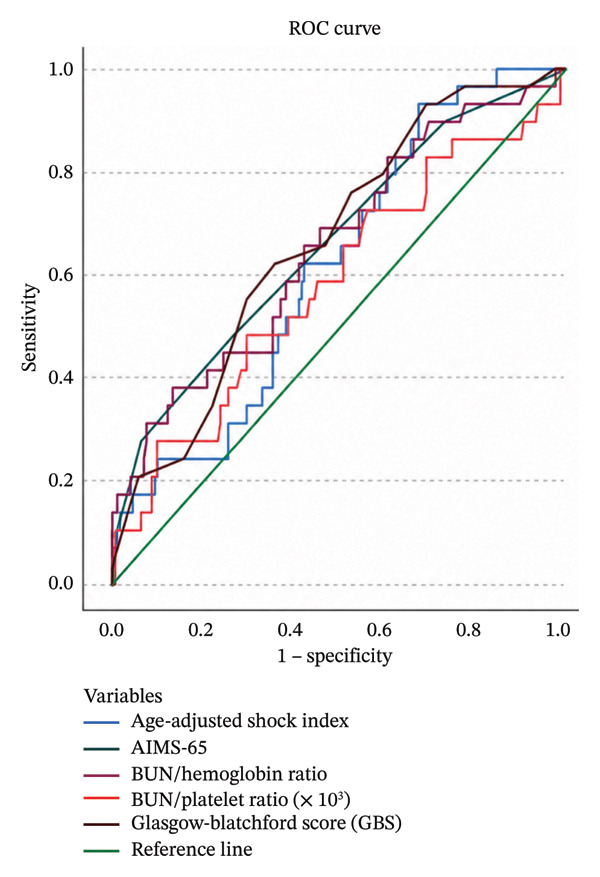
ROC analysis of parameters according to ICU admission.

The performance of the BUN/haemoglobin ratio in predicting ICU admission was evaluated using ROC analysis, yielding an AUC of 0.656 and an optimal cut‐off value of > 7.175. At this threshold, sensitivity was 37.93%, specificity 86.86%, PPV 32.35% and NPV 87.3% (Figure 3, Table 4).

**TABLE 4:** ROC‐derived cut‐off values and predictive performance of study parameters for in‐ICU admission.

	AUC	Youden’s index threshold	Sensitivity (%)	Specificity (%)	Accuracy (%)	NPV (%)	PPV (%)
Age‐Adjusted Shock Index	0.620	≥ 41.51	93.10	33.14	41.67	96.6	17.9
BUN/Haemoglobin Ratio	0.656	≥ 7.175	37.93	86.86	79.90	89.6	33
BUN/Platelet Ratio (× 10^3^)	0.587	≥ 0.194	48.28	70.86	67.65	89.5	22.6
AIMS‐65	0.666	≥ 11.5	48.28	73.71	70.10	89.58	23.33
Glasgow–Blatchford Bleeding Score	0.667	≥ 1.5	64.57	64.57	64.22	91.13	22.50

When the ASI was assessed for predicting ICU admission, ROC analysis produced an AUC of 0.620, indicating moderate discriminatory ability. The optimal cut‐off value was > 41.51, with a sensitivity of 93.10% and a specificity of 33.10% (Figure 3, Table 4).

For the BUN/platelet ratio, ROC analysis yielded an AUC of 0.587, suggesting limited discriminatory performance for ICU admission. The optimal cut‐off value identified from the ROC curve was > 0.19, at which the balance between sensitivity and specificity was maximised (Figure 3, Table 4).

In the analysis of ICU admission, the AIMS‐65 score had an AUC of 0.666, with a sensitivity of 48.28%, specificity of 73.71%, accuracy of 70.10%, NPV of 89.58% and PPV of 23.33%. The Glasgow–Blatchford score had an AUC of 0.667, with a sensitivity of 64.57%, specificity of 64.57%, accuracy of 64.22%, NPV of 91.13% and PPV of 22.50% (Figure 3, Table 4).

**FIGURE 4:**
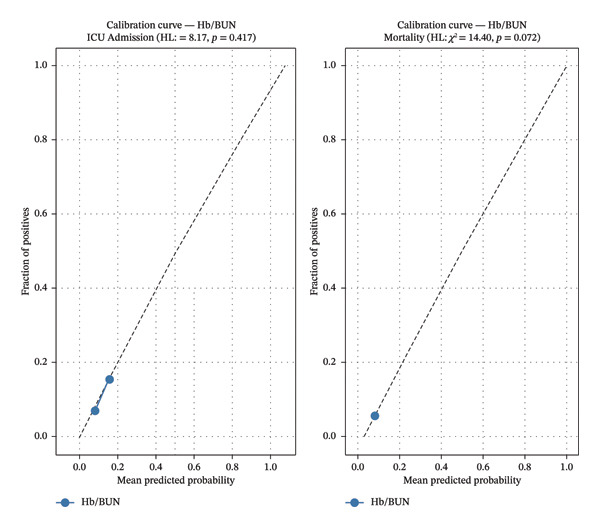
Calibration curves of the Hb/BUN ratio for predicting ICU admission and in‐hospital mortality.

The Hb/BUN ratio showed good calibration for ICU admission (Hosmer–Lemeshow *χ* ^2^ = 8.17, *p* = 0.417). Bootstrap‐corrected AUC was 0.704 (95% CI: 0.623–0.809) and cross‐validated AUC was 0.676 (Figure 4). DCA results are presented in Supporting Information (see Supporting Information).

**FIGURE 5:**
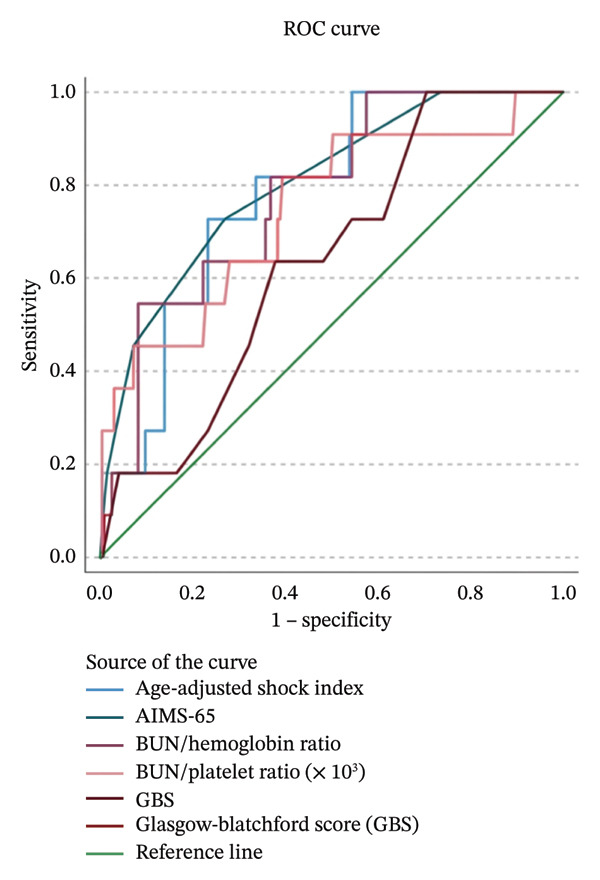
ROC analysis of parameters according to mortality.

ROC analyses showed that the ASI, the BUN/haemoglobin ratio and the BUN/platelet ratio were each significantly associated with in‐hospital mortality. For the BUN/haemoglobin ratio, a cut‐off value of > 8.45 demonstrated good predictive performance for mortality (AUC: 0.779); at this threshold, sensitivity was 54.55%, specificity 91.71%, PPV 27.2%, and NPV 97.26% (Figure 5, Table 5).

**TABLE 5:** ROC‐derived cut‐off values and predictive performance of study parameters for in‐hospital mortality.

	AUC	Youden’s index threshold	Sensitivity (%)	Specificity (%)	Accuracy (%)	NPV (%)	PPV (%)
Age‐Adjusted Shock Index	0.780	≥ 61.72	72.73	76.68	76.47	90.71	45.45
BUN/Haemoglobin Ratio	0.779	≥ 8.45	54.55	91.71	89.71	87.79	64.86
BUN/Platelet Ratio (× 10^3^)	0.747	≥ 0.17	81.82	60.62	61.76	92.54	36.36
AIMS‐65	0.803	≥ 1.5	72.73	73.06	73.04	97.92	13.33
Glasgow–Blatchford Bleeding Score	0.638	≥ 7.5	100	29.53	33.33	100.00	7.48

For the BUN/platelet ratio, a cut‐off value of > 0.17 also showed good discriminatory ability for mortality (AUC: 0.747); sensitivity was 72.73%, specificity 60.82%, PPV 10.56%, and NPV 98.32% at this threshold. For ASI, the AUC was 0.780, with a sensitivity of 72.73%, a specificity of 76.68%, a PPV of 14.93% and an NPV of 98.03 (Figure 5, Table 5).

In the analysis of in‐hospital mortality, the AIMS‐65 score had an AUC of 0.803, with a sensitivity of 72.73%, specificity of 73.06%, accuracy of 73.04%, NPV of 97.92%, and PPV of 13.33%. The Glasgow–Blatchford score had an AUC of 0.638, with a sensitivity of 100%, specificity of 29.53%, accuracy of 33.33%, NPV of 100%, and PPV of 7.48% (Figure 5, Table 5).

The Hb/BUN ratio demonstrated adequate calibration for mortality (Hosmer–Lemeshow *χ* ^2^ = 14.40, *p* = 0.072). Bootstrap‐corrected AUC was 0.849 (95% CI: 0.760–0.954) and cross‐validated AUC was 0.805 (Figure 4). DCA demonstrated a net benefit of the full model over treat‐all and treat‐none strategies at clinically relevant threshold probabilities (see Supporting Information).

In multivariable logistic regression analysis, the BUN/haemoglobin ratio was identified as an independent predictor of ICU admission (OR 1.18, 95% CI 1.06–1.33, *p* = 0.004). The ASI was not independently associated with ICU admission (*p* = 0.341). The overall model demonstrated moderate discriminative ability with an AUC of 0.666. The DeLong analysis showed that AIMS‐65 had a significantly higher AUC than GBS (ΔAUC = 0.165, 95% CI 0.017–0.314, *p* = 0.029), and BUN/hemoglobin also demonstrated a significantly higher AUC compared to GBS (ΔAUC = 0.141, *p* = 0.018), whereas no statistically significant differences were observed between the other parameters (all *p* > 0.05). No multicollinearity was detected among the variables included in the model (all VIF values < 2). Internal validation using bootstrap resampling (5000 iterations) confirmed model stability (see Supporting Information).

## 4. Discussion

This study evaluated the prognostic value of parameters derived from laboratory and vital signs obtained at presentation in patients with UGIB. The main advantage of these parameters is that they are rapid, inexpensive and easily calculable at the time of admission. In contrast, conventional scoring systems (such as the Glasgow–Blatchford and Rockall scores) include more complex components. Moreover, variables such as heart rate, systolic blood pressure, BUN, creatinine and haemoglobin can not only be measured quickly but also provide continuous quantitative data, thereby assisting in risk stratification in terms of bleeding severity and the need for intervention. Recent evidence suggests that combining simple physiological and biochemical parameters, such as NEWS‐2 and AIMS65, may enhance early risk stratification in UGIB patients and complement traditional scoring systems in emergency settings [15].

In the study by Doğru et al., the shock index demonstrated stronger predictive performance for mortality and prognosis at 30, 180 and 360 days compared with AIMS65, Glasgow–Blatchford, and Rockall scores [16]. Other studies have shown that the ASI better predicts in‐hospital mortality and adverse outcomes in UGIB patients compared with the shock index and modified shock index [17, 18]. In the study by Wierzchowski et al., which compared UGIB patients aged ≥ 75 and < 75 years, rebleeding rates were higher and prognosis worse in the older group. Differences in aetiology, comorbidities and antiplatelet use suggested that standard approaches may be insufficient in elderly patients with UGIB [19].

In our study, ASI was significantly associated with both mortality and ICU admission. Its high NPV indicated that patients with a low ASI had a low risk of mortality. In a UK study including 6750 cases, the median age of UGIB patients was reported as 68 years (range 16–100) [20]. Given that the UGIB population is predominantly elderly, the concept of reduced physiological reserve and frailty beyond chronological age becomes clinically relevant. In this context, ASI could reflect not only haemodynamic instability but also diminished physiological reserve by integrating age and circulatory status. Its ease and rapid calculation in emergency settings could facilitate early risk stratification, particularly in geriatric and frail populations, and its integration into standard assessment could support timely intervention and individualised management decisions.

In the study by Tomizawa et al., a BUN threshold of 21.0 mg/dL showed high specificity (93.0%) but low sensitivity (36.4%) for distinguishing upper from lower gastrointestinal bleeding [21]. While elevated values strongly support UGIB, normal values may miss many cases. Beyond diagnostic discrimination, BUN may also reflect the biochemical burden and indirectly the clinical severity of bleeding. This is consistent with the underlying physiology. A graded relationship exists between decreasing haemoglobin levels and increasing bleeding severity. While a threshold of 10.8 g/dL has been used to indicate the presence of UGIB, values below 8 g/dL suggest severe bleeding requiring urgent intervention (likelihood ratio: 4.5–6.2) [22, 23]. Lower haemoglobin levels in patients with BUN ≥ 21 mg/dL support the association of severe bleeding with both increased blood loss (low haemoglobin) and intraluminal protein digestion and urea absorption (high BUN) [24]. This pathophysiological link suggests that evaluating BUN together with haemoglobin may provide more meaningful information than either parameter alone. Indeed, Yeşil et al. demonstrated that a high urea/haemoglobin ratio was positively correlated with the likelihood of active bleeding requiring urgent endoscopic therapy [25]. Similarly, elevated BUN levels have been associated with ICU admission, rebleeding and mortality [26].

In our ROC analysis for hospitalised patients, the high NPV of the BUN/haemoglobin ratio indicated that low‐risk patients could be safely managed on the ward. In mortality analyses, the BUN/haemoglobin cut‐off of > 8.45 demonstrated good predictive performance. The median BUN/haemoglobin ratio was approximately 2.5‐fold higher in nonsurvivors compared with survivors (8.47 vs 3.43), consistent with literature linking high BUN and low haemoglobin levels to adverse outcomes [27, 28]. However, the low number of mortality events represents a limitation, and the BUN/haemoglobin ratio has not yet been validated as an independent predictor. Therefore, our findings should be considered as hypothesis‐generating, suggesting that this simple ratio may practically reflect prognostic information.

In the study by Huang et al., thrombocytopenia was more strongly associated with UGIB than with lower gastrointestinal bleeding [29]. The BUN/platelet ratio may theoretically reflect the coexistence of massive or life‐threatening bleeding and impaired coagulation mechanisms [30]. An elevated BUN/platelet ratio may simultaneously represent increased bleeding severity (via elevated BUN) and reduced haemostatic reserve (via decreased platelet count), warranting careful interpretation, particularly in patients with marked thrombocytopenia. Evaluating platelet count together with BUN may provide a more comprehensive prognostic assessment by reflecting both the metabolic and haematological aspects of bleeding.

In our study, although the BUN/platelet ratio showed a borderline association with mortality, it was not significantly associated with ICU admission. Its acceptable discriminative performance for mortality (AUC: 0.747) and high NPV at low thresholds suggest potential utility in ruling out low mortality risk. However, the limited number of mortality events (*n* = 11) reduces the stability and generalisability of AUC and cut‐off estimates; therefore, these findings should be interpreted as hypothesis‐generating and require validation in larger, preferably externally validated cohorts.

For ICU admission, the discriminative performance of the BUN/platelet ratio was limited; however, a cut‐off value of < 0.17 yielded a high NPV, supporting ward‐level management of low‐risk patients. Notably, platelet count is not included in commonly used UGIB risk scoring systems; thus, the BUN/platelet ratio may provide complementary prognostic information not captured by existing tools.

The parameters evaluated in our study reflect different aspects of patient status: ASI represents haemodynamic instability, the BUN/haemoglobin ratio reflects acute blood loss and metabolic response, and the BUN/platelet ratio reflects both bleeding severity and haematologic reserve. We found only a moderate correlation (*r* ≈ 0.44) between ASI and BUN‐based ratios.

These findings should be interpreted with caution, and the evaluated parameters are not intended to replace established risk scoring systems but rather to serve as complementary tools in early emergency department assessment. However, the absence of multivariable analysis limits the ability to determine their independent prognostic contributions beyond shared haemodynamic and metabolic determinants. Future studies could explore the development of an alternative risk score integrating these three parameters. Nevertheless, the inclusion of all eligible patients identified during the predefined study period and the statistical optimisation of cut‐off values using ROC analysis constitute important strengths of the study.

## 5. Limitations

The limitations of this study include its single‐centre and retrospective design, which may increase the risk of selection bias and measurement errors. The relatively low number of outcome events—particularly mortality (*n* = 11) and ICU admissions (*n* = 29)—may reduce statistical power, limit the stability of ROC curve analyses, and lead to variability in AUC estimates and cut‐off values, thereby restricting generalisability. External or temporal validation using an independent cohort was not feasible due to the single‐centre retrospective design. Therefore, the calibration and discrimination metrics reported in this study reflect internal validation only and should be interpreted cautiously with respect to generalisability to other populations. For this reason, multivariable regression analysis could not be performed for mortality, and the independent prognostic contribution of the evaluated parameters for this outcome could not be determined. Consequently, the findings should be interpreted as exploratory and hypothesis‐generating rather than definitive.

The evaluated parameters were compared with established scoring systems such as the Glasgow–Blatchford score and AIMS‐65; however, the exclusion of patients with malignancy limited the applicability of the Rockall score within this cohort. In addition, the NPVs and selected cut‐off thresholds reported in this study may vary across different populations, as they are influenced by event prevalence. Finally, the study focused solely on short‐term outcomes, such as in‐hospital mortality and ICU admission, limiting interpretation regarding longer‐term clinical outcomes, including rebleeding or 30‐day mortality.

## 6. Conclusion

There are limited studies in the literature evaluating the relationship between the parameters examined in our study and UGIB. The most notable common feature of these three parameters is their high NPV for adverse outcomes. In particular, each index was highly effective in identifying low‐risk patients who were unlikely to require intensive care. Our findings suggest that ASI and the BUN/haemoglobin ratio may be useful for initial risk assessment and triage in emergency settings, particularly as complementary tools rather than replacements for established scoring systems such as the Glasgow–Blatchford and Rockall scores. Future multicentre prospective studies may enable the integration of these ratios into risk stratification algorithms.

## Funding

No specific grant or financial support was received for this research from any funding agency in the public, commercial, or not‐for‐profit sectors.

## Ethics Statement

Ethics approval for this study was obtained from the Scientific Research Ethics Committee of Bezmialem Vakif University (approval number: 2025/388; meeting date: October 22, 2025; decision number: 16). Due to the retrospective observational nature of the study and the use of anonymized patient data, the requirement for informed consent was waived by the ethics committee.

## Conflicts of Interest

The authors declare no conflicts of interest.

## Supporting Information

Additional supporting information can be found online in the Supporting Information section.

## Supporting information


**Supporting Information** Supporting Information 1. Multivariable logistic regression model for ICU admission, including regression coefficients, odds ratios, and 95% confidence intervals. Supporting Information 2. Pairwise comparisons of ROC curve AUCs using the DeLong method. Supporting Information 3. Multicollinearity assessment of variables included in the multivariable model using variance inflation factor (VIF) and tolerance statistics. Supporting Information 4. Decision curve analysis (DCA) demonstrating the clinical utility of the Hb/BUN ratio model for predicting ICU admission and in‐hospital mortality. Supporting Information 5. Completed TRIPOD checklist for transparent reporting of the study in accordance with TRIPOD recommendations.

## Data Availability

The data supporting the findings of this study are not publicly available due to patient privacy restrictions but are available from the corresponding author upon reasonable request.
