# Supplementary material for: Predicting Adverse Outcomes in Upper Gastrointestinal Bleeding: A Focus on Blood Urea Nitrogen‐Based Ratios and Age‐Adjusted Shock Index
Source: Emerg Med Int. 2026 Jul 8;2026:3772312. doi: 10.1155/emmi/3772312 (PMC13342697; doi:10.1155/emmi/3772312)
Supplement: Supplementary file 1 — Supporting Information Supporting Information 1. Multivariable logistic regression model for ICU admission, including regression coefficients, odds ratios, and 95% confidence intervals. Supporting Information 2. Pairwise comparisons of ROC curve AUCs using the DeLong method. Supporting Information 3. Multicollinearity assessment of variables included in the multivariable model using variance inflation factor (VIF) and tolerance statistics. Supporting Information 4. Decision curve analysis (DCA) demonstrating the clinical utility of the Hb/BUN ratio model for predicting ICU admission and in‐hospital mortality. Supporting Information 5. Completed TRIPOD checklist for transparent reporting of the study in accordance with TRIPOD recommendations. [file EMMI-2026-3772312-s001.zip › Supp Material.docx]

**Supplementary Material 1: Multivariant Logistic Regression**

Model Coefficients – ICU Admission

### 95% Confidence Interval

| **Predictor** | **Estimate** | **SE** | **Z** | **p** | **Odds ratio** | **Lower** | **Upper** |
| --- | --- | --- | --- | --- | --- | --- | --- |
| Intercept | -3.18604 | 0.57798 | -5.512 | <.001 | 0.0413 | 0.0133 | 0.128 |
| BUN/Haemoglobin Ratio | 0.16942 | 0.05864 | 2.889 | 0.004 | 1.1846 | 1.0560 | 1.329 |
| Age-Adjusted Shock Index | 0.00935 | 0.00982 | 0.953 | 0.341 | 1.0094 | 0.9902 | 1.029 |

*Not.* Estimates represent the log odds of "ICU = 2" vs. "Ward = 1"

**Supplementary Material 2: Pairwise comparison of AUCs between variables using the DeLong method**

| **Comprasion** | **AUC Differance** | **%95 GA** | **z** | **p** |
| --- | --- | --- | --- | --- |
| AIMS-65 vs Glasgow-Blatchford Bleeding Score | 0.165 | 0.017–0.314 | 2.187 | 0.029 |
| AIMS-65 vs Age-Adjusted Shock Index | 0.022 | −0.134–0.179 | 0.280 | 0.779 |
| AIMS-65 vs BUN/Haemoglobin Ratio | 0.024 | −0.110–0.159 | 0.353 | 0.724 |
| Glasgow-Blatchford Bleeding Score vs Age-Adjusted Shock Index | −0.143 | −0.298–0.012 | −1.803 | 0.071 |
| Glasgow-Blatchford Bleeding Score vs BUN/Haemoglobin Ratio | −0.141 | −0.258–−0.024 | −2.369 | 0.018 |
| Age-Adjusted Shock Index vs BUN/Haemoglobin Ratio | 0.002 | −0.132–0.136 | 0.028 | 0.978 |

**Supplementary Material 3:** Multicollinearity

| **Variables** | **VIF** | **Tolerance** |
| --- | --- | --- |
| BUN/Haemoglobin Ratio | 1.19 | 0.839 |
| Age-Adjusted Shock Index | 1.17 | 0.856 |

**Supplementary Material 4:** Decision curve analysis of Hb/BUN ratio for predicting ICU admission and mortality.

**
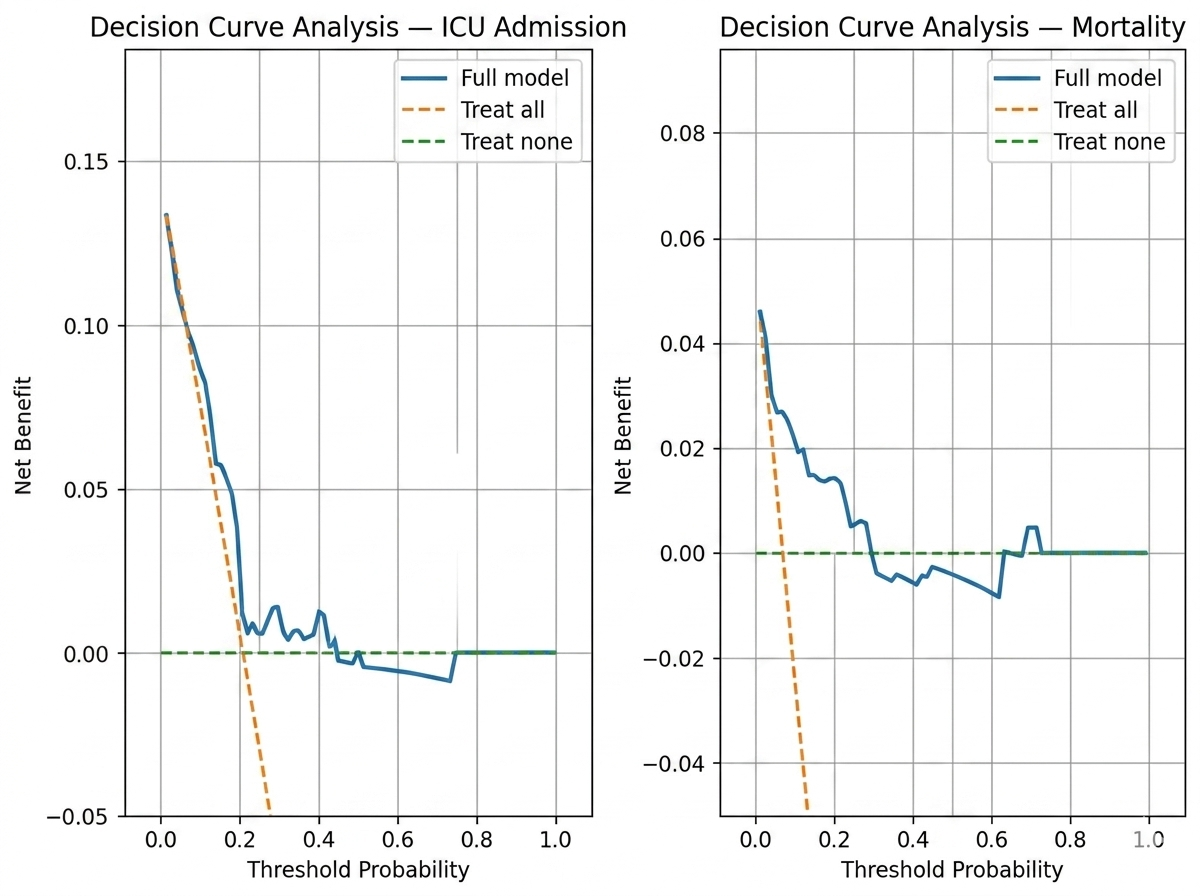
**
