# Supplementary material for: Predicting Adverse Outcomes in Upper Gastrointestinal Bleeding: A Focus on Blood Urea Nitrogen‐Based Ratios and Age‐Adjusted Shock Index
Source: Emerg Med Int. 2026 Jul 8;2026:3772312. doi: 10.1155/emmi/3772312 (PMC13342697; doi:10.1155/emmi/3772312)
Supplement: Supplementary file 1 — Supporting Information Supporting Information 1. Multivariable logistic regression model for ICU admission, including regression coefficients, odds ratios, and 95% confidence intervals. Supporting Information 2. Pairwise comparisons of ROC curve AUCs using the DeLong method. Supporting Information 3. Multicollinearity assessment of variables included in the multivariable model using variance inflation factor (VIF) and tolerance statistics. Supporting Information 4. Decision curve analysis (DCA) demonstrating the clinical utility of the Hb/BUN ratio model for predicting ICU admission and in‐hospital mortality. Supporting Information 5. Completed TRIPOD checklist for transparent reporting of the study in accordance with TRIPOD recommendations. [file EMMI-2026-3772312-s001.zip › TRIPOD_Checklist_Filled.docx]

**TRIPOD Checklist: Prediction Model Development**

*Predicting Adverse Outcomes in Upper Gastrointestinal Bleeding: A Focus on Blood Urea Nitrogen Based Ratios and Age-Adjusted Shock Index*

| **Item** | **Checklist Item** | **Page No.** | **Relevant Text from Manuscript** |
| --- | --- | --- | --- |
| **TITLE AND ABSTRACT** | | | |
| **1** | Identify the study as developing and/or validating a multivariable prediction model, the target population, and the outcome to be predicted. | 1 | Title: 'Predicting Adverse Outcomes in Upper Gastrointestinal Bleeding: A Focus on Blood Urea Nitrogen Based Ratios and Age-Adjusted Shock Index.' Target population: adult ED patients with UGIB. Outcomes: mortality and ICU admission. Development study. |
| **2** | Provide a summary of objectives, study design, setting, participants, sample size, predictors, outcome, statistical analysis, results, and conclusions. | 1 | Abstract covers all required elements: retrospective cohort, single centre, n=204, 1 Jan–31 Dec 2022, predictors (BUN/Hb, BUN/platelet, ASI), outcomes (mortality 5.4%, ICU 14%), ROC analysis, AUC values, NPVs, and clinical conclusions. |
| **INTRODUCTION** | | | |
| **3a** | Explain the medical context and rationale for developing or validating the multivariable prediction model, including references to existing models. | 2 | Introduction describes limitations of GBS, AIMS-65 and Rockall scores and rationale for novel BUN-based ratios and ASI as complementary prognostic parameters in UGIB. References to existing models provided (refs 12–14). |
| **3b** | Specify the objectives, including whether the study describes development or validation of the model, or both. | 2 | 'We evaluated the association of the ASI, BUN/haemoglobin and BUN/platelet ratios, as well as AIMS-65 and the GBS with mortality and ICU admission.' This is a model development study; no external validation was performed. |
| **METHODS** | | | |
| **4a** | Describe the study design or source of data separately for the development and validation data sets, if applicable. | 3 | 'This study was conducted as a single-centre retrospective observational cohort study.' Single dataset used for development. Internal validation performed via bootstrap (5000 iterations) and 10-fold cross-validation. No separate external validation dataset. |
| **4b** | Specify key study dates, including start and end of accrual and, if applicable, end of follow-up. | 3 | Accrual: 1 January 2022 to 31 December 2022. Follow-up: in-hospital (outcomes ascertained at discharge or death). |
| **5a** | Specify key elements of the study setting, including number and location of centres. | 3 | Single centre: emergency department, Bezmialem Vakif University, Istanbul, Turkey. |
| **5b** | Describe eligibility criteria for participants. | 3 | Inclusion: adults ≥18 years presenting to ED with UGIB (confirmed by clinical presentation, ICD-10 coding, and endoscopic findings). Exclusion: oesophageal variceal bleeding, lower GI sources, trauma, pregnancy, malignancy, prior diagnosis at another institution, incomplete data. |
| **5c** | Give details of treatments received, if relevant. | — | Not applicable. This is a prognostic study; treatment allocation was not a study variable. |
| **6a** | Clearly define the outcome that is predicted, including how and when assessed. | 3 | Two outcomes: (1) in-hospital mortality; (2) ICU admission. Both ascertained retrospectively from hospital electronic records at the end of hospital stay. |
| **6b** | Report any actions to blind assessment of the outcome to be predicted. | — | Retrospective study design; formal blinding was not feasible. Outcomes ascertained from pre-existing electronic records independently of predictor values. |
| **7a** | Clearly define all predictors used, including how and when they were measured. | 3 | Predictors: BUN/Hb ratio, BUN/platelet ratio, Age-adjusted shock index (ASI = shock index × age), AIMS-65 score, Glasgow-Blatchford score. All measured at ED presentation using routine admission laboratory and vital sign data. |
| **7b** | Report any actions to blind assessment of predictors for the outcome and other predictors. | — | Retrospective data collection; formal blinding not feasible. Predictors derived from objective laboratory and vital sign measurements. |
| **8** | Explain how the study size was arrived at. | 4 | 'No formal a priori sample size calculation based on outcome events was performed, as this was a retrospective cohort study including all eligible patients within the predefined study period.' The limited number of events (mortality n=11, ICU n=29) is acknowledged as a limitation. |
| **9** | Describe how missing data were handled. | 3 | Two patients with incomplete data were excluded during screening (complete-case analysis). No imputation was performed. The included cohort (n=204) had no further missing data. |
| **10a** | Describe how predictors were handled in the analyses. | 4 | All predictors treated as continuous variables in ROC and regression analyses. Optimal cut-off values derived from ROC curve analysis (Youden index). Cut-offs reported for clinical use. |
| **10b** | Specify type of model, all model-building procedures (including predictor selection), and method for internal validation. | 4 | Binary logistic regression. Variables selected based on univariable significance (p<0.05) and clinical relevance. Events-per-variable principle applied to avoid overfitting. Box-Tidwell procedure for linearity; VIF for multicollinearity. Internal validation: bootstrap (5000 iterations, BCa CIs) and 10-fold cross-validation. |
| **10d** | Specify all measures used to assess model performance and, if relevant, to compare multiple models. | 4 | Discrimination: AUC with 95% CI; DeLong method for pairwise AUC comparisons; IDI and NRI vs. AIMS-65. Calibration: Hosmer-Lemeshow test (χ², p-value); calibration curves. Overall performance: Brier score. Clinical utility: Decision Curve Analysis (Supplementary Material). Cross-validated AUC reported. |
| **11** | Provide details on how risk groups were created, if done. | 5–7 | Risk groups (low vs. high risk) defined using ROC-derived cut-off values for each predictor. Sensitivity, specificity, PPV and NPV reported at each threshold (Tables 4 and 5). |
| **RESULTS** | | | |
| **13a** | Describe the flow of participants through the study, including the number with and without the outcome. | 3–5 | 274 patients screened; 70 excluded; 204 included. ICU admission: n=29 (14%); no ICU: n=175 (86%). Mortality: n=11 (5.4%); survivors: n=193 (94.6%). Flow diagram: Figure 1. |
| **13b** | Describe the characteristics of the participants, including the number with missing data for predictors and outcome. | 5 | Table 1 presents baseline demographics, comorbidities, presenting symptoms, vital signs and laboratory values. Median age 70 years; 66% male. 2 patients with missing data excluded before analysis; no further missing data in included cohort. |
| **14a** | Specify the number of participants and outcome events in each analysis. | 5 | All 204 participants included in ROC analyses. Multivariable logistic regression for ICU admission: n=204 (29 events). Mortality analysis: n=204 (11 events) — multivariable analysis not feasible due to insufficient events. |
| **14b** | If done, report the unadjusted association between each candidate predictor and outcome. | 5–7 | Tables 2 and 3 report median values with IQR for each predictor by outcome group (ICU vs. ward; survivors vs. non-survivors) with Mann-Whitney U p-values. ROC AUCs with sensitivity/specificity reported in Tables 4 and 5. |
| **15a** | Present the full prediction model to allow predictions for individuals (regression coefficients, intercept or baseline survival). | 7 | Multivariable logistic regression for ICU admission: BUN/Hb ratio OR 1.18 (95% CI: 1.06–1.33, p=0.004); ASI OR not statistically significant (p=0.341). Full model AUC: 0.704 (ICU), 0.849 (mortality — bootstrap-corrected). |
| **15b** | Explain how to use the prediction model. | 5–7 | Cut-off values provided for each predictor (e.g., BUN/Hb >8.45 for mortality, >7.175 for ICU admission). Clinical interpretation of NPVs explained in Results and Discussion sections. |
| **16** | Report performance measures (with CIs) for the prediction model. | 5–7 | AUC with 95% CI (bootstrap): mortality 0.849 (0.760–0.954); ICU 0.704 (0.623–0.809). CV-AUC: mortality 0.805; ICU 0.676. HL test: mortality χ²=14.40, p=0.072; ICU χ²=8.17, p=0.417. Calibration curves: Figure 5. IDI and NRI reported vs. AIMS-65. Brier scores reported. |
| **DISCUSSION** | | | |
| **18** | Discuss any limitations of the study (nonrepresentative sample, few events per predictor, missing data). | 9–10 | Limitations section discusses: single-centre retrospective design (selection bias risk), low event numbers (mortality n=11; ICU n=29) limiting multivariable analysis for mortality, absence of external validation, and restriction to in-hospital outcomes. |
| **19b** | Give an overall interpretation of the results, considering objectives, limitations, results from similar studies, and other relevant evidence. | 7–9 | Discussion contextualises findings against published literature on ASI, BUN-based ratios and platelet count in UGIB. Findings described as 'hypothesis-generating'. Parameters recommended as complementary tools rather than replacements for established scores. |
| **20** | Discuss the potential clinical use of the model and implications for future research. | 9–10 | 'Our findings suggest that ASI and the BUN/haemoglobin ratio may be useful for initial risk assessment and triage in emergency settings, particularly as complementary tools.' Future multicentre prospective studies recommended to enable integration into risk stratification algorithms. |
| **OTHER INFORMATION** | | | |
| **21** | Provide information about the availability of supplementary resources, such as study protocol, Web calculator, and data sets. | — | Bootstrap validation results presented in Supplementary Material. Decision Curve Analysis provided in Supplementary Material. Data available from corresponding author upon reasonable request. |
| **22** | Give the source of funding and the role of the funders for the present study. | 11 | 'No specific grant or financial support was received for this research from any funding agency in the public, commercial, or not-for-profit sectors.' |

We recommend using the TRIPOD Checklist in conjunction with the TRIPOD Explanation and Elaboration document.
